# Relationship Between the Choice of Clinical Treatment, Gait Functionality and Kinetics in Patients With Comparable Knee Osteoarthritis

**DOI:** 10.3389/fbioe.2022.820186

**Published:** 2022-03-11

**Authors:** Simone Tassani, Laura Tio, Francisco Castro-Domínguez, Jordi Monfort, Juan Carlos Monllau, Miguel Angel González Ballester, Jérôme Noailly

**Affiliations:** ^1^ BCN MedTech, DTIC, Universitat Pompeu Fabra, Barcelona, Spain; ^2^ IMIM, Barcelona, Spain; ^3^ Rheumatology Department, Hospital del Mar, Barcelona, Spain; ^4^ Orthopedic Surgery and Traumatology Department, Hospital del Mar, Barcelona, Spain; ^5^ ICREA, Barcelona, Spain

**Keywords:** Knee osteoarthritis (KOA), gait, confounding adjustment, multifactorial analysis, functionality

## Abstract

**Objective:** The objective of this study was to investigate the relationship between the choice of clinical treatment, gait functionality, and kinetics in patients with comparable knee osteoarthritis.

**Design:** This was an observational case-control study.

**Setting:** The study was conducted in a university biomechanics laboratory.

**Participants:** Knee osteoarthritis patients were stratified into the following groups: clinical treatment (conservative/total knee replacement (TKR) planned), sex (male/female), age (60–67/68–75), and body mass index (BMI) (<30/≥30). All patients had a Kellgren–Lawrence score of 2 or 3 (N = 87).

**Main Outcome Measures:** All patients underwent gait analysis, and two groups of dependent variables were extracted:

• Spatiotemporal gait variables: gait velocity, stride time, and double-support time, which are associated with patient functionality.

• Kinetic gait variables: vertical, anterior–posterior, and mediolateral ground reaction forces, vertical free moment, joint forces, and moments at the ankle, knee, and hip. Multifactorial and multivariate analyses of variance were performed.

**Results:** Functionality relates to treatment decisions, with patients in the conservative group walking 25% faster and spending 24% less time in the double-support phase. However, these differences vary with age and are reduced in older subjects. Patients who planned to undergo TKR did not present higher knee forces, and different joint moments between clinical treatments depended on the age and BMI of the subjects.

**Conclusions:** Knee osteoarthritis is a multifactorial disease, with age and BMI being confounding factors. The differences in gait between the two groups were mitigated by confounding factors and risk factors, such as being a woman, elderly, and obese, reducing the variability of the gait compression loads. These factors should always be considered in gait studies of patients with knee osteoarthritis to control for confounding effects.

## Introduction

The definition of objective criteria for total knee replacement (TKR) remains a matter of debate, leading to different rates of surgery in different countries (Ghomrawi et al., 2014). The clinician–patient relationship (O’Neill et al., 2007) and their expectations and beliefs about conservative therapy and surgery (O’Brien et al., 2019) are likely to influence the final treatment; therefore, it is difficult to find objective criteria to help the decision.

Osteoarthritis (OA) is more prevalent in women (4.8%) than in men (2.8%) and increases with age and obesity (Cross et al., 2014). Therefore, sex, body mass index (BMI), and age play a direct role in the progression of the disease and are possible sources of confounding (Kyriacou and Lewis, 2016). The variation of the Kellgren–Lawrence (KL) grade is often used to define the radiological progression of knee osteoarthritis (KOA) (Braun and Gold, 2012). However, the pain experienced by patients is not directly related to KL grade (Schiphof et al., 2013). Therefore, it is still unclear whether the KL grade or the functionality of the patients, in terms of pain, discomfort, or capability to perform daily activities, should guide the treatment decision of KOA in terms of TKR or conservative management.

KOA core treatment is nonpharmacological (e.g., exercise and weight loss). If it is necessary, pharmacological treatment must be chosen carefully considering the patient profile, e.g., nonsteroidal anti-inflammatory agents, symptomatic slow-acting drugs for osteoarthritis (SYSADOAs), or hyaluronic acid infiltrations (Author Anonymous, 2000).

Beyond a certain point of OA progression, these treatments are ineffective, and clinical decisions switch to TKR. Nonetheless, different rates of TKR have been described in different countries (Gómez-Barrena et al., 2014; Gunsche et al., 2020). These data highlight the importance of identifying homogeneous indication criteria for TKR. Objective treatment decisions should be an index of KOA progression (Astephen et al., 2008) and might be reflected by specific gait functionality and kinetics that are unavoidably altered along the course of the disease. However, translating such a mental exercise into practical recommendations may not be straightforward.

The literature pointed out that macro factors, such as obesity, can directly affect the microlevel development of the pathology (Felson et al., 1988; Coggon et al., 2002; Berenbaum et al., 2013; Wluka et al., 2013). If sex, age, and weight have any effect on OA, they should be studied simultaneously in a multifactorial analysis to detect possible nonlinearities in the assessment of KOA. This analysis should be performed taking into consideration the fact that the same factors also affect gait. In fact, sex (McKean et al., 2007), age (Paterson et al., 2009; Park et al., 2016) BMI (Messier et al., 1996; De Souza et al., 2005; Dufek et al., 2012) and the stage of OA (Wang et al., 2010; De Pieri et al., 2019) influence gait, and it is difficult to understand which factors are dependent and independent. As a result, there are no defined objective criteria for selecting TKR (Gómez-Barrena et al., 2014).

Accordingly, an attempt was made to investigate whether any relation exists between the treatment decision and gait functionality and kinetics. The study was performed in a prospective clinical cohort of KOA patients with similar KL grades. Sex, age, and BMI were considered to verify possible interactions and confounding effects (Kyriacou and Lewis, 2016) in the treatment selection through a multifactorial and multivariate analysis. It was hypothesized that patients undergoing TKR have less gait functionality, higher loads, and higher moments at the joints.

## Materials and Methods

The present work presents a case control study in which patients with similar KL levels are stratified based on the clinical decision whether to undergo TKR and on other clinically relevant factors as possible sources of confounding. The analysis was performed prior to intervention.

### Patient Recruitment

The clinical histories of all patients diagnosed with KOA at the Rheumatology/Orthopaedic Surgery Department of Hospital del Mar, Barcelona, Spain, were revised prospectively to build a proper cohort (eligibility criteria in Table 1). Selected patients were asked to follow a wash-up treatment for a period of 3 months for intra-articular hyaluronic acid infiltrations, 2 months for any SYSADOA, 1 month for oral or intra-articular corticoids, and 1 week for nonsteroidal anti-inflammatory drugs or opioid drugs. The study followed the Good Clinical Practice guidelines and the Declaration of Helsinki, and the Clinical Research Ethical Committee approved the protocol (2016/6747/I). All participants signed an approved informed consent.

**TABLE 1:** Patient eligibility criteria.

Inclusion criteria
Male and Females aged between 60 and 75 years (both included)
Fulfilment of the American College of Rheumatology (ACR) classification criteria for clinical Knee OA
Presence of radiographic OA in the knee joint, scored as 2 or 3 according to Kellgren and Lawrence (KL) definition
Presence of symptomatology (pain, dysfunction and/or effusion) in the last 3 months
Ability to provide written informed consent
Exclusion Criteria
Need for assistance or support to walk (crutch, walker)
Present OA either in the lateral femorotibial compartment or in the patellofemoral compartment exclusively
Secondary OA
Partial or total meniscectomy
Inflammatory or connective tissue diseases
Overuse of the joint from work or sporting activities
Pathological varus or valgus deformity
Underlying health condition
Uncompensated diseases
Fibromyalgia
Presence of microcrystals in the articular space
MRI contraindications
Abuse substances use in the 6 months prior to the study

The recruitment was performed according to the clinical treatment as follows: a conservative group, that included patients with a diagnosis of KOA attending to follow-up clinical visits at any of the two Departments and following namely any nonsurgical procedure, such as pharmacological treatment or healthier lifestyle modifications (exercise, weight loss, etc.) or a TKR-planned group, that involved patients enrolled in the waiting list for a TKR surgery. To avoid confusion by secondary relevant factors, sex, age (categorized as 60–67 and 68–75 years old), and BMI (categorized as nonobese, BMI <30, and obese, BMI 30 or higher) were also considered in the recruitment. This approach enables the identification of the effect of each factor on gait, as well as their interactions.

All patients had a KL score of 2 or 3 where grade 2 (minimal) presents definite osteophytes and possible joint space narrowing while grade 3 (moderate) presents moderate multiple osteophytes, definite narrowing of joint space and some sclerosis and possible deformity of bone ends. KL was controlled and matched as a possible source of confounding by indication (Kyriacou and Lewis, 2016).

### Gait Analysis

**FIGURE 1:**
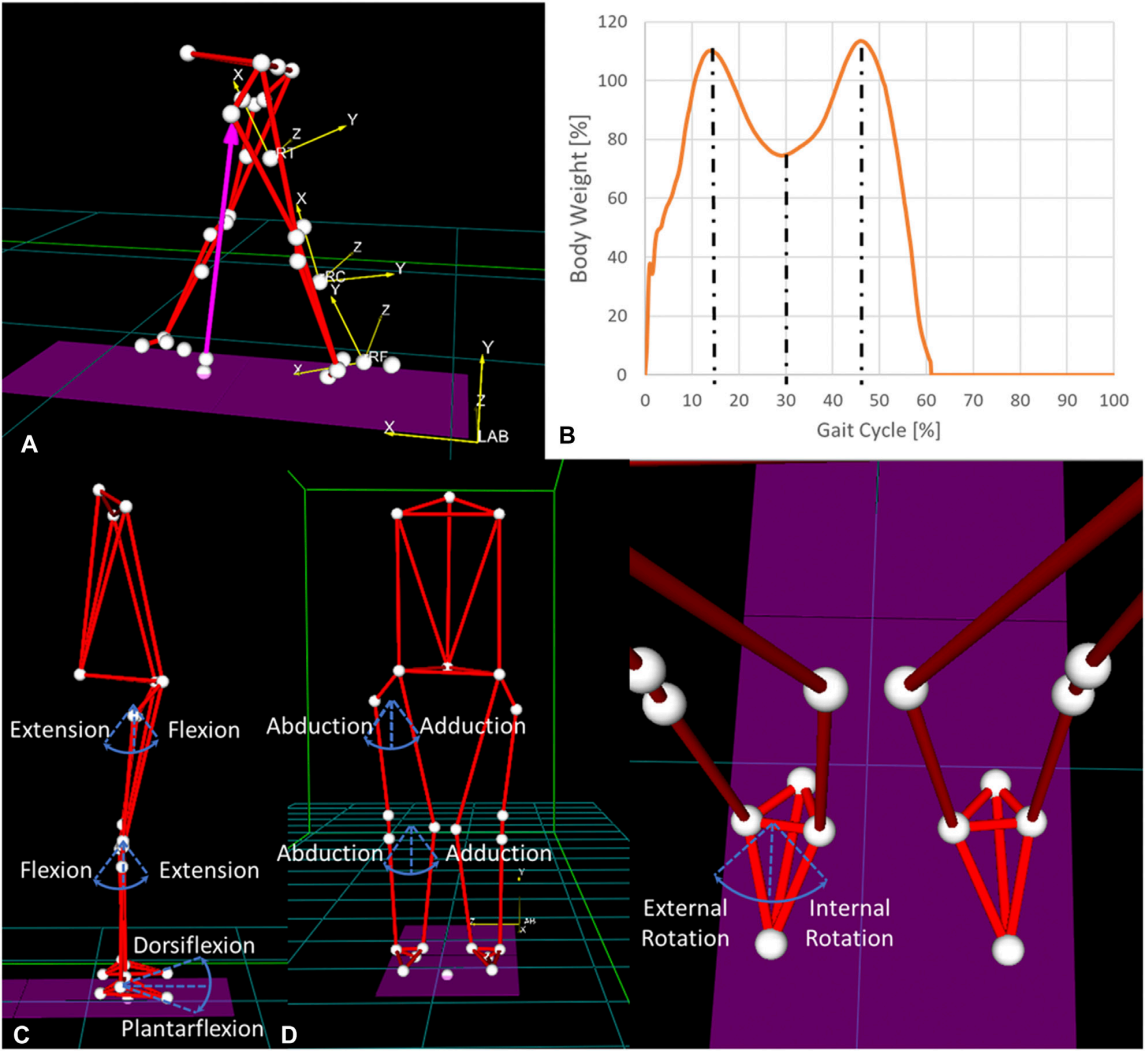
**(A)** Reference systems of the laboratory and of the right thigh, calf, and foot and **(B)** vertical ground reaction force during a gait cycle. The three dashed lines identify the three points of analysis. **(C)** Articular rotations in the sagittal plane, **(D)** articular rotations in the coronal plane, and **(E)** articular rotations in the transverse plane.

Gait analysis was performed using eight cameras (1.5 Mpixel, 250 fps; BTS Smart-DX 700, BTS Engineering, Milan, Italy) and two force plates (500-Hz sampling; BTS P-6000, BTS Engineering, Milan, Italy). The Helen Hayes marker protocol with medial markers was used (Davis et al., 1991). Briefly, the protocol consists of 22 reflective markers (Figures 1C,D), three markers for trunk (one marker in correspondence to the 7th cervical vertebra, one by the right acromion and one by the left acromion), pelvis (one marker on each ASIS and one marker in the second sacral vertebra), thighs (one marker on the lateral femoral condyle, and one on the medial femoral condyle and the last one placed on the lateral portion of the thigh in the great trochanter area), shank (one marker on the lateral malleolus one on the medial malleolus and one placed directly on the lateral portion of the shank in the region of the fibula’s head) and two markers for each foot (one marker in the space between the heads of the second and third metatarsals and one on the heel). At first a static acquisition was performed, and medial markers were removed prior to perform the gait sequences. Each subject was asked to perform a minimum of five valid gait sequences over a 10-m catwalk at a self-selected speed. The volunteer had a free walk of at least 3 m before stepping over two force plates. Human gait has intrinsic variability; however, sometimes single gait trials can be visually very different from the average. For this reason, a visual consistency evaluation was performed over valid walking trials. Only gaits trials that showed no discrepancies were included in the analysis (see Supplementary Materials). At the end of this process, the parameters computed in each trial were normalized over one gait cycle. Kinetics variables were also normalized over the body weight. Finally, the 5 gait trials of each subject were arithmetically averaged, and a second-level analysis was carried out throughout the study.

Gait analysis allowed the extraction of two groups of dependent variables.

#### Spatiotemporal gait variables

The velocity of gait was computed as the ratio of the distance between the two heel strikes to the time needed to perform a gait cycle. The first heel strike of each leg was identified using the ground reaction force registration while the second one was identified using the 3D visualization of the markers of the foot during gait. Video recording and force plate recording were already synchronized during capture, avoiding discrepancies in the measurement. The time of a stride and the percentage of time spent in double support were also recorded as indices of functionality.

#### Kinetic gait variables

Vertical, anterior–posterior, and mediolateral ground reaction forces were extracted from the force plates. The vertical free moment was also extracted. An inverse dynamic analysis was performed, and the results were projected onto joint coordinate systems (Figure 1A; Table 2). Joint contact forces and internal moments were computed using Smart Analyzer (BTS Engineering, Milan, Italy) at the ankle, knee, and hip of the osteoarthritic leg. They included compression, mediolateral and anterior–posterior shear forces, and flexion–extension, abduction–adduction, and internal-external rotation moments at hip and knee. Ankle dorsi–plantar flexion moments and medio-lateral forces were also computed (Figures 1C–E).

**TABLE 2:** Definition of the directions of each force and moment used in the study.

Force	Component of the reference system
Hip Medio-Lateral shear (HPML)	Component Z of the reference of the pelvis
Hip Compression (HPCP)	Component X of the reference of the thigh
Hip Anterior-Posterior (HPAP)	Vector product of the HPML and HPCP
Knee Medio-Lateral shear (KML)	Component Z of the reference of the thigh
Knee Compression (KCP)	Component X of the reference of the calf
Knee Anterior-Posterior (KAP)	Vector product of KML and KCP
Ankle Medio-Lateral (AML)	Component Z of the reference of the calf
**Moment**	**Component of the reference system**
Hip Flex-Extension (HPFE)	Component Z of the reference of the pelvis
Hip Intra-Extra (HPIE)	Component X of the reference of the thigh
Hip Abduction-Adduction (HPAA)	Vector product of the HPFE and HPIE
Knee Flex-Extension (KFE)	Component Z of the reference of the thigh
Knee Intra-extra (KIE)	Component X of the reference of the calf
Knee Abduction-Adduction (KAA)	Vector product of KFE and KIE
Ankle Dorsi-Plantar Flexion (ADPF)	Component Z of the reference of the calf

For each patient, force and moment values were normalized to body weight and analyzed at the percentage of the gait cycle where the derivative of the vertical ground reaction force was zero, thereby identifying three time points (Figure 1B).

### Design of Experiment

Recruited patients were grouped based on clinical treatment, age, BMI, and sex, as previously described. Each factor had two levels; therefore, the entire analysis comprised 16 groups. Three multivariate and multifactorial analyses of variance (MANOVA) were performed: one for the spatiotemporal parameters and two for the kinetic parameters (Bonferroni correction *p* < 0.018). The power analysis for the principal effects, assuming an average effect size of 0.15, suggests sample sizes of 96, 64, and 64 participants to obtain actual powers of 0.84, 0.90, and 0.95, respectively, for spatiotemporal and kinetic analyses.

To explore possible confounding effects further, different covariates were included in the analysis: height for the spatiotemporal analysis, and stride time and velocity for the force and moment analyses. The analysis at three different time points led to include time as a “within factor” comparing the variations of force and moment along the analyzed gait (repeated measure analysis).

Whenever one of the factors was found to be significant, a univariate ANOVA was performed to identify the significant variables. Significance was adjusted over the number of dependent variables tested through Bonferroni correction (spatiotemporal *p* < 0.018, forces *p* < 0.005, moments *p* < 0.006). Analyses were performed using SPSS (version 23.0; IBM Corp., Armonk, NY, United States) for the osteoarthritic leg.

## Results

### Descriptive Results

Eighty-seven subjects were recruited (global powers 0.78, 0.99, and 0.99) and stratified into 16 groups derived based on the combination of the four factors of the analysis (Table 3). Seventy-two subjects presented with bilateral KOA. The remaining 15 were split into conservative (8) and TKR (7) to maintain a balanced analysis. Male patients belonging to the TKR-planned group were particularly difficult to recruit.

**TABLE 3:** Distribution of the recruited subjects over the 16 groups.

Sex	Clinical treatment	Age	BMI	N
Male	Conservative	60–67	Nonobese	5
Male	Conservative	60–67	Obese	6
Male	Conservative	68–75	Nonobese	6
Male	Conservative	68–75	Obese	6
Male	TKR-planned	60–67	Nonobese	2
Male	TKR-planned	60–67	Obese	1
Male	TKR-planned	68–75	Nonobese	3
Male	TKR-planned	68–75	Obese	7
Female	Conservative	60–67	Nonobese	7
Female	Conservative	60–67	Obese	6
Female	Conservative	68–75	Nonobese	6
Female	Conservative	68–75	Obese	6
Female	TKR-planned	60–67	Nonobese	6
Female	TKR-planned	60–67	Obese	7
Female	TKR-planned	68–75	Nonobese	6
Female	TKR-planned	68–75	Obese	7

### Spatiotemporal Functionality

**FIGURE 2:**
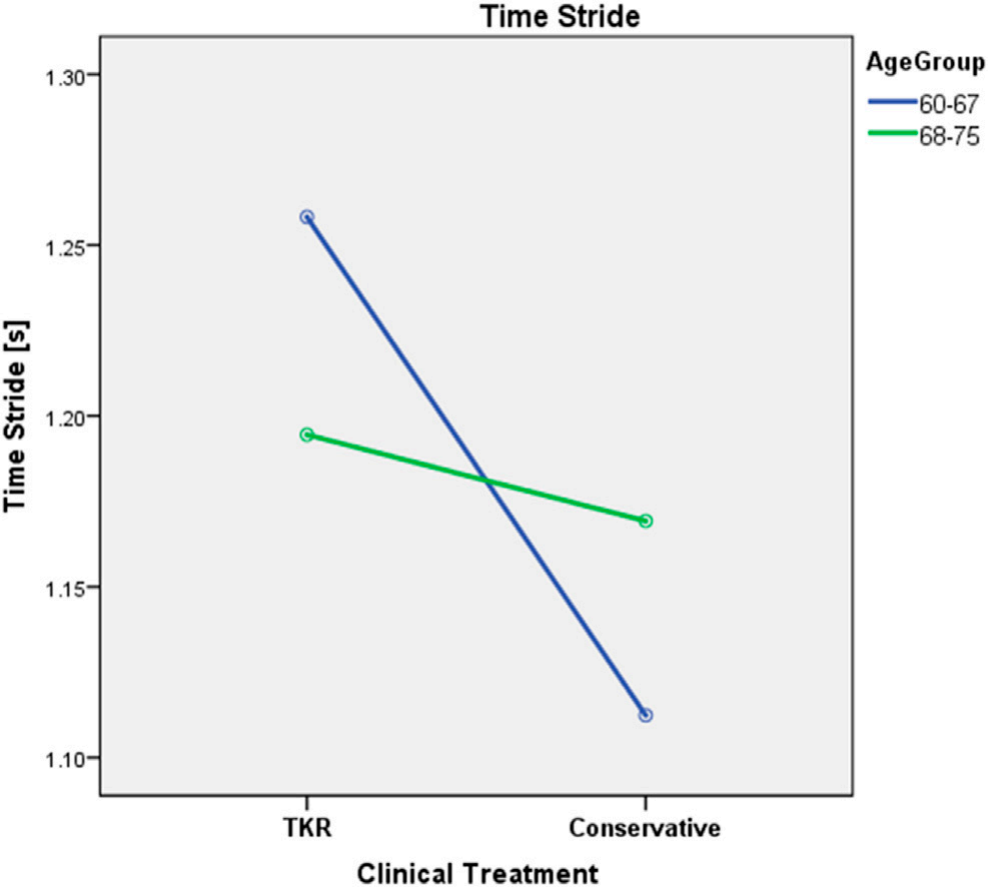
Effect of the interaction between clinical treatment and age over mean stride time (*p* = 0.006).

All residuals showed a normal distribution. The averages and standard deviations for the four main factors are reported in Table 4, together with the significance of the main factors and the list of significant interactions. Height was a significant covariate (*p* = 0.007, Supplementary Material), suggesting the influence of subject height on stride, double-support times, and velocity. Functionality appears to be related to age and clinical treatment. TKR-planned patients needed more time to take a step (8.52% increase), spent more time in the double-stand position (31.94%), and walked more slowly (19.92%). Age was also a significant factor with a multivariate effect. However, its dependence seemed to vary with treatment, as shown by the interaction between clinical treatment and age. In particular, the time required to make a stride varied depending on the interaction between the two factors. Figure 2 suggests that subjects from the conservative group needed less time for a stride, but younger subjects had greater variability than older subjects.

**TABLE 4:** Functional Averages and standard deviation for functional parameters. Yellow color indicates significant multivariate effect. Green color indicates significant univariate effect. *p* < 0.018.

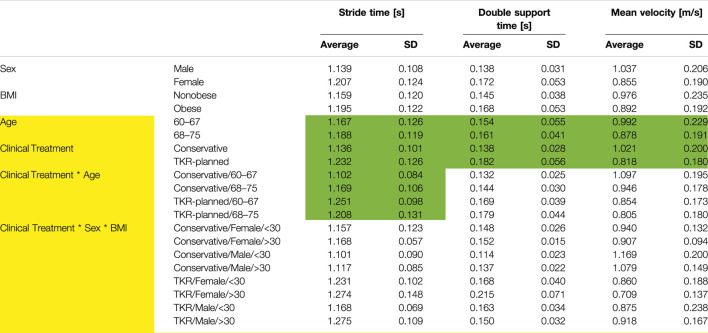

### Kinetics

#### Analysis of Forces

**FIGURE 3:**
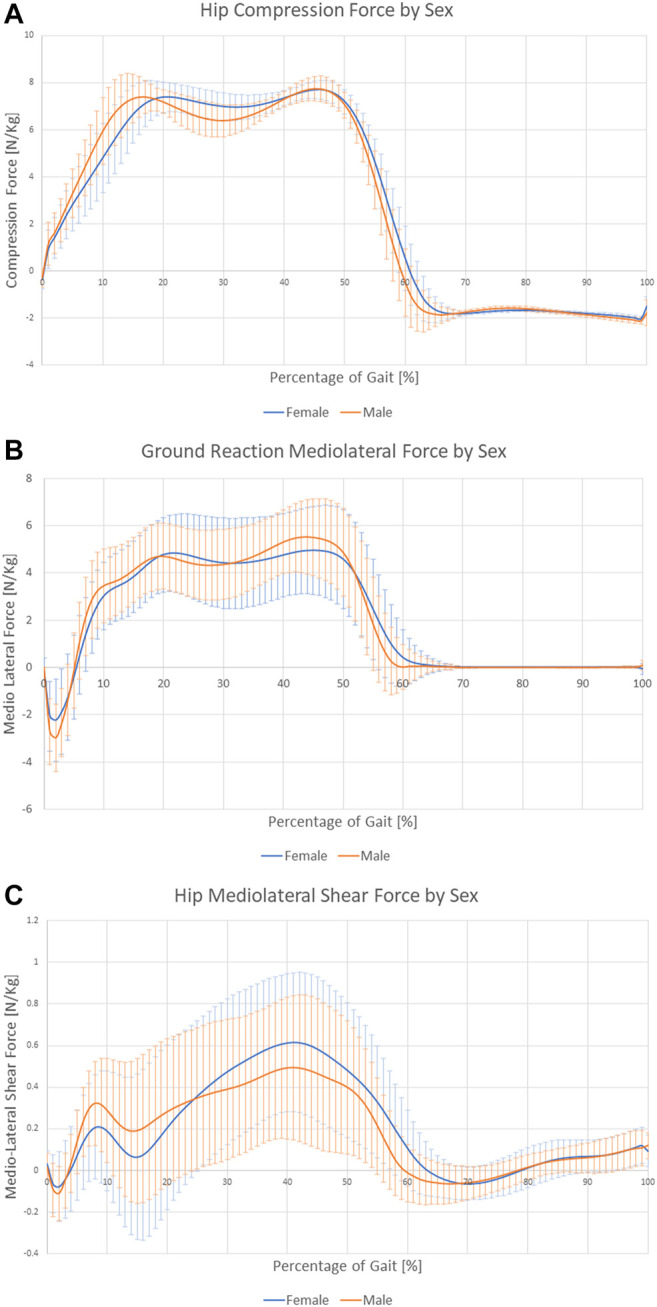
Forces compared between the two sexes: **(A)** hip compression force profile, **(B)** ground reaction mediolateral force, and **(C)** hip mediolateral shear force.

**FIGURE 4:**
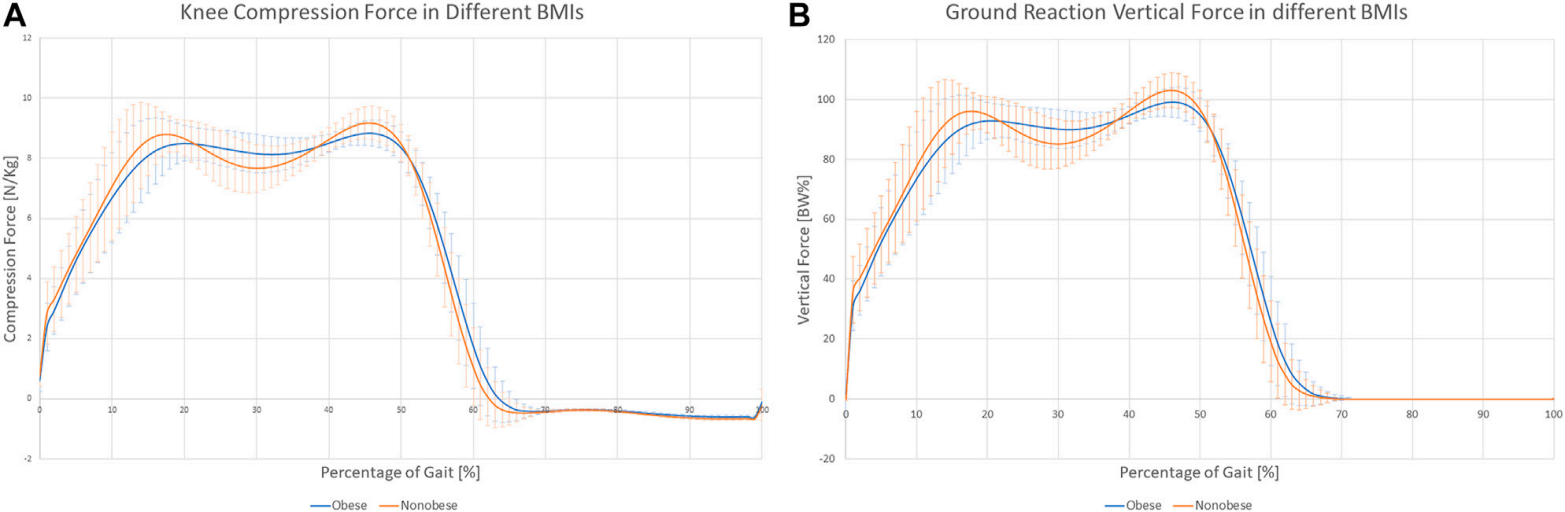
Knee compression **(A)** and ground reaction **(B)** force profiles for Obese and Nonobese subjects.

Clinical treatment was not related to the forces experienced during gait. Sex was the only factor that affected the forces (Table 5). Females showed slightly higher compression forces at the hip, as averaged over the three time points, even if forces were normalized by body weight, and inertial factors were considered as covariate. However, the test for repeated measures over the three time points confirmed a significant influence of time, but only for some of the analyzed forces (Table 5). This dependence was further influenced by sex and BMI. Figure 3 shows the different variations in the load profile over time in men and women. In particular, Figure 3A shows that, whereas peak loads are similar between males and females in the hip, the minimum load is higher in women, resulting in reduced load variations from heel strike to toe off. As for the interaction between time and BMI, the knee compression force showed higher peak loads and larger variations along gait in nonobese versus obese subjects (Figure 4A). The results were repeated at ground level (Figure 4B).

**TABLE 5:** Forces means and standard deviation. Yellow color indicates significant multivariate effect (*p* < 0.018). Green color indicates significant univariate effect. (*p* < 0.005). Vertical (VE), Anterior-Posterior (AP) and Medio-Lateral (ML) ground reaction (GR) forces, respectively GRVE, GRAP and GRML. Joints forces were computed at the Ankle (A), Knees (K) and Hips (HP) of osteoarthritic leg and were referred as compression (CP), Medio-Lateral (ML) and Anterior-Posterior (AP) shear, respectively AML, KCP, KML, KAP, HPCM, HPML, HPAP.

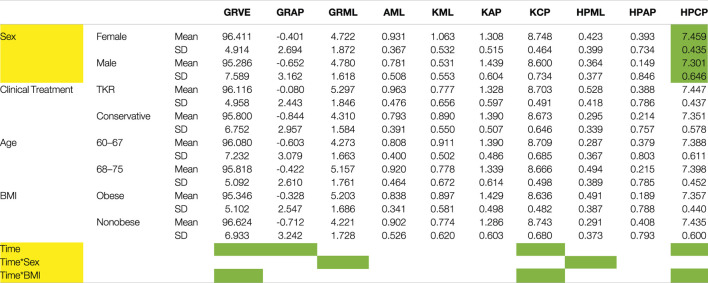

Complete descriptions of the averages and standard deviations for the four main factors over the three time points are reported in the Supplementary Material.

#### Analysis of Joint Moments

**FIGURE 5:**
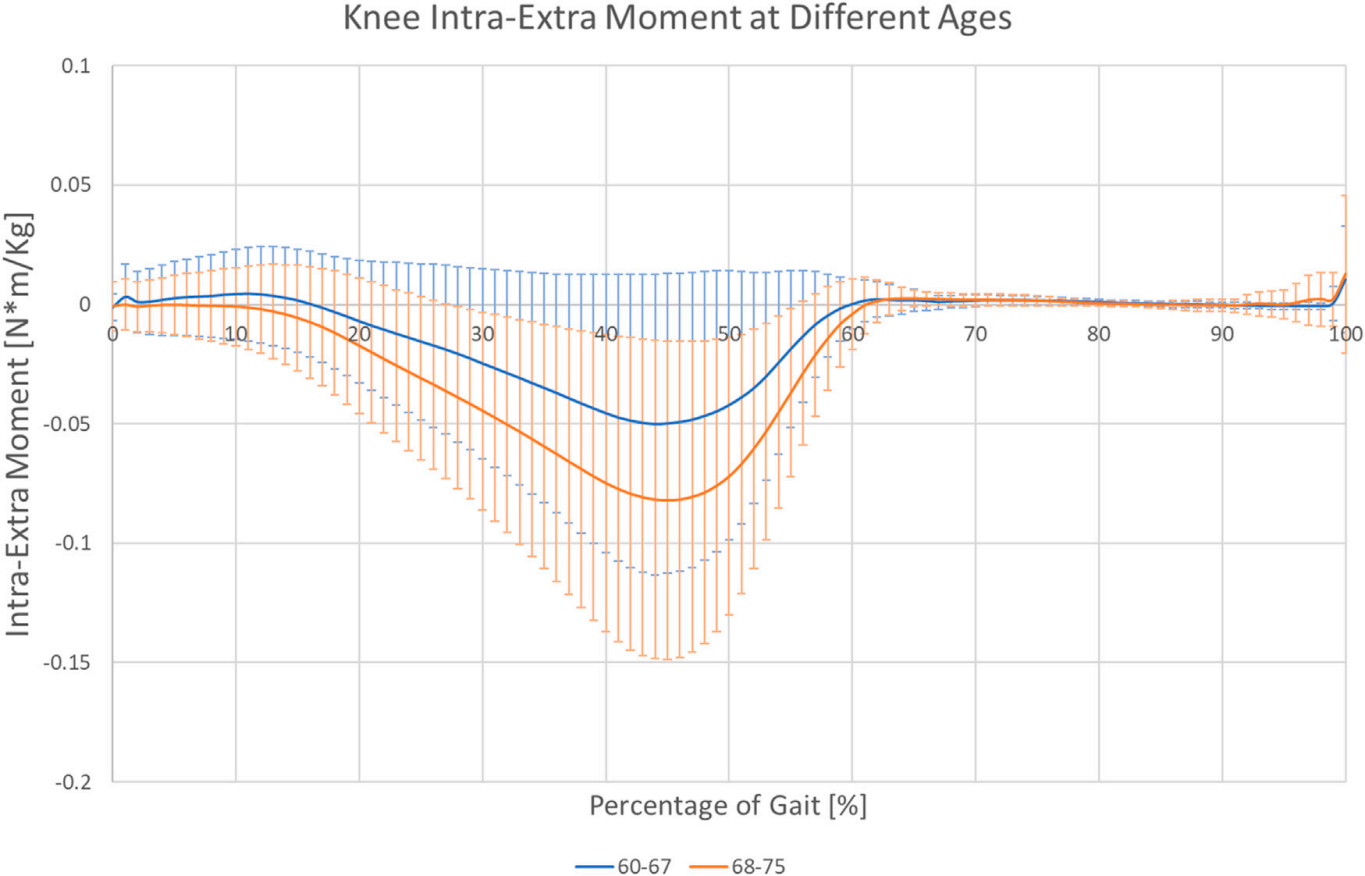
Knee internal-external rotation moment profile for 60–67- and 68–75-year-old subjects.

**FIGURE 6:**
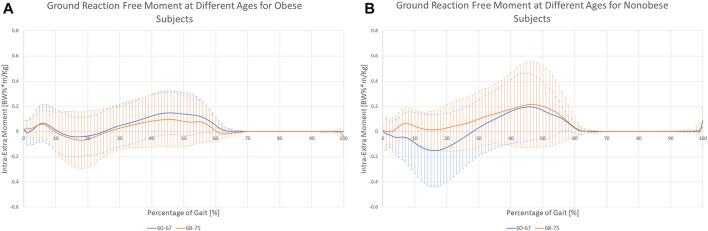
Variation of the ground reaction free moment for the two age groups while considering Obese **(A)** or Nonobese **(B)**.

**FIGURE 7:**
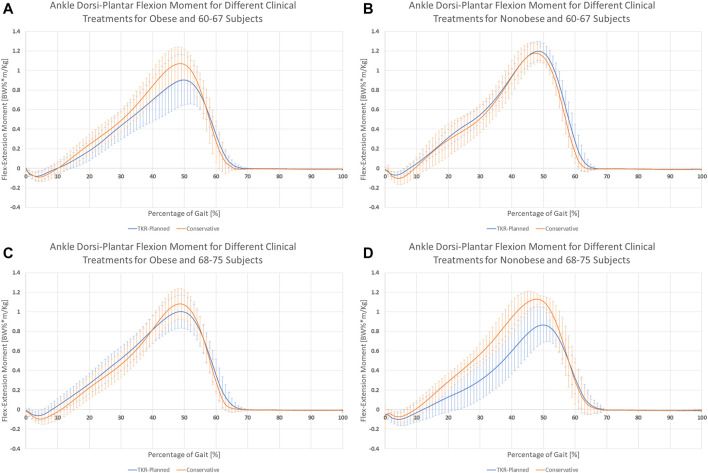
Interaction between clinical treatment, BMI, and age over ankle dorsi-plantar flexion. Behavior differences between the two clinical treatment are visible in **(A)** obese subjects with age range 60–67 and **(D)** nonobese subjects with age range 68–75. In **(B)** nonobese subjects age 60–67 and **(C)** obese subjects age 68–75, no significant differences are noticeable between the two clinical treatments.

The clinical treatment chosen by the patients was not related directly to the joint moments experienced during gait (Table 6). However, joint moments are correlated with age. Younger patients showed, on average, approximately half of the values of the older subjects (Figure 5; Table 6). A significant interaction between age and BMI has also been reported. In obese subjects, age did not affect the moment profile along gait (Figures 6A), while significant differences between ages were reported for nonobese subjects (Figures 6B). Interactions among clinical treatment, age, and BMI were reported, as illustrated by the ankle dorsiflexion moment profiles in the different combinations of subjects (Figure 7). Conservative subjects showed a higher peak of ankle moment in the presence of obesity and an age range of 60–67 (Figures 7A) and in nonobese subjects aged 68–75 (Figures 7D) but not in the other combinations. Higher-level interactions among the four factors were also observed.

**TABLE 6:** Moments means and standard deviation. Yellow color indicates significant multivariate effect (*p* < 0.018). Green color indicates significant univariate effect. (*p* < 0.006). Ground reaction free moment (GRFM), Flexion-Extension (FE), Abduction-Adduction (AA) and Internal-External rotation (IE) moments are reported at knee and hip of osteoarthritic leg, respectively KFE, KAA, KIE and HPFE, HPAA, HPIE. Dorsi-Plantar Flexion moment of the ankle is also reported (ADPF).

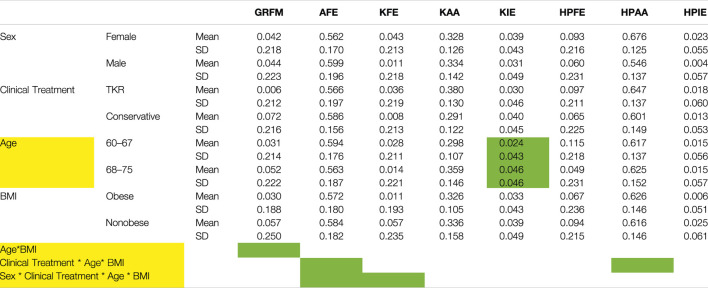

Complete descriptions of averages and standard deviations for the four main factors over the three time points are reported in the Supplementary Material.

## Discussion

TKR is the “gold-standard” treatment for patients with severe symptomatic KOA who have failed nonsurgical management and suffer significant impairment in their quality of life. Nevertheless, the proportion of people with unfavorable long-term outcomes ranges from 10 to 34% (Beswick et al., 2012)*.* TKR decisions are still conditioned by a high degree of susceptibility related to patient’s and doctor’s thoughts and beliefs. Although the analysis of gait measurements in OA might provide valuable objective biomarkers, previous studies have failed to achieve consensus (Mills et al., 2013). Such difficulty can be partially explained by the presence of confounding effects (Kyriacou and Lewis, 2016) and the lack of multifactorial analyses designed to understand such effects. In the present study, an attempt was made to achieve a better integrated vision of KOA patients and their gait by considering four different factors and three covariates in a cohort of 87 subjects. The final aim of this study was to identify objective criteria for TKR decisions.

### Recruitment

Subject recruitment to achieve balanced groups was challenging, showing the commonly reported higher prevalence of KOA in women (Buckwalter and Lappin, 2000). The men who were difficult to recruit belonged to three groups of TKR treatment. The total number of candidates examined at the Hospital del Mar, Barcelona, Spain, for the TKR-planned group exceeded 500 per year, so the outcome of the recruitment suggests that epidemiologically, within the inclusion criteria of this study, the combination of TKR-planned/male/obese/elderly is the only one with a prevalence similar to that of women.

### Multifactorial Manova

Multifactorial ANOVA and MANOVA are powerful statistical tools which have many potentialities but also some limitations. Researchers must be aware of both in order to perform a good analysis of the results.

Multifactorial analysis allows to perform statistical evaluation of several factors using a single test. Moreover, the stratification of patients in several subgroups allows to analyze also the interactions among more factors permitting the evaluation of non-linearities. However, the power of the test is not constant among all the factors and interaction analyzed. In the present study four main factors are presented i.e., clinical treatment, sex, BMI and age. These four factors are tested at the maximum power allowed by the test since for each of them all the patients are divided only in two groups (39 TKR-planned against 48 Conservative, 36 male against 51 female, 40 subjects with age 60–67 against 47 with age 68–75, 46 obese and 41 non obese). This approach allows to verify effects of the analyzed factor, independently by the presence of other factors. In the present study the power obtained for the two analyses of kinetic data testify a high power of the test related to the main factors. The computed power of the spatiotemporal MANOVA was 0.78, which is slightly less than the standard value of 0.8. This might reflect in 2% higher probability of type II error. Multifactorial analysis allows also to evaluate the effect of interaction among factors; however, researchers must consider that the dimension of the groups is decreased as the level of the interaction increase. This means that, while first level interactions can still be considered robust results since performed over four groups of about 20 subjects each, higher level interactions must be considered as preliminary results to be confirmed in future works. In particular, the limited number of TKR-planned men in three of the studied groups is expected to introduce imbalance in the study when high-level interactions, including sex, are analyzed. Hence, results limited to the interactions of clinical treatment–sex–age and clinical treatment–sex–age–BMI should be considered as preliminary.

Finally, when multifactorial MANOVA results are presented, standard deviations can be very high because they include all the analyzed subjects. However, the analyses considered the effect of each separated factor, therefore significant factors can be trusted to be significant despite the showed high standard deviation.

### Gait

The evaluation of gait results must be performed taking into consideration that statistical tests cannot identify causation but only relation among factors. The common nomenclature of dependent and independent variables is used only to discriminate between fixed factors and measured variables.

Identifiers of functionality were found to depend on the clinical treatment. Subjects selected for TKR presented a less functional joint because they required more time for a stride, walked more slowly, and spent more time in double support than subjects selected for conservative treatment.

The correlation between time for a stride and severity of OA is one of the few strong evidences in the literature (Chen et al., 2003; Astephen et al., 2008; Zeni and Higginson, 2009). The results related to the correlation between the variation in gait velocity and the severity of OA are not so clear (Heiden et al., 2009; Butler et al., 2011). According to the results, these apparent contradictions might be related to the lack of inclusion of height as a covariate or to the absence of age as a factor of analysis. In fact, older subjects seemed to be less affected by velocity variations. Finally, TKR-planned subjects spent a higher percentage of time in double support, showing a gait characterized by different proportions of single- and double-support phases. This can be related to pain or fear of pain, as subjects perceive the double-support phase as a secure position (Haddas et al., 2018). However, the reported differences were not constant over the years. In fact, while younger subjects require less time for stride than elders in the conservative group, this relation might be inverted in TKR-planned subjects (Figure 2; Table 4, *p* = 0.006). Due to the unquestionable interactions between gait and clinical data (such as pain, function or emotional variables), the future study of their association may allow to link clinical situations directly to the gait characteristics of the subject.

Subjects waiting for TKR do not apply a higher mechanical load on their joints or higher joint moments, thus rejecting the original hypothesis. Sex was the only main factor showing a relationship with the analyzed forces. Moreover, the effect is related to time and presents different patterns. Men seemed to have larger variations in hip compression and mediolateral ground reaction forces (Figures 3A,B), and women showed higher variability of mediolateral shear forces at the hip (Figure 3C). Finally, analysis of BMI confirmed the importance of inertial effects, showing greater variation in nonobese subjects. This variation was consistent at the ground, knee, and hip levels (Figure 4 and Supplementary Results). Joint moments apparently correlated with the clinical treatment, but its impact depended on BMI, age (Figure 7), and sex (Supplementary Material). Therefore, differences between groups were visible only under specific combinations of factors.

Age also had a direct effect on moments during gait. Elderly patients had higher absolute moment values (Table 6), suggesting an effect of the change in posture during aging. Age also showed a significant interaction with BMI in the MANOVA (*p* = 0.011)*.* Figure 6 shows that differences in the ground reaction free moment are minimal between the two age groups for obese subjects but become relevant in nonobese patients (*p* = 0.018), suggesting an increased variability related to low BMI.

The three points analysis over the time of gait also allowed to perform a screening of variables variability along gait. Time was supposed to be a significant variable since the values in analyses were selected in three representative points of the gait. However, only four forces presented a significant relation with time and no moments showed significant time dependency. This was not expected and make the analysis of the variability an interesting point to explore.

Variations in gait patterns presented in this study might be related to pain or stiffness, but a longitudinal study would be required to identify specific causal relationships.

### Limitations

The presented study has some limitations.

The grouping of subjects by clinical treatment is assumed to be correct. This can sometimes be misleading, and a minority of subjects might be incorrectly selected, thereby increasing the noise in the analysis.

Spatiotemporal analysis did not consider swing and stance because they always sum up to 100% of the gait cycle and lead to the cancelation of variability in the multivariate analysis, moreover the used variables can present some relation among them, which is not suggested for MANOVA analysis, however this would not affect the subsequent univariate analysis which can therefore confirm the results of the MANOVA.

Contact forces computed as a result of inverse dynamics are considered to be the minimum necessary to maintain dynamic equilibrium in the system. Muscle co-contraction is a known issue in OA subjects (Thoma et al., 2016) that can introduce relevant variations in the compression forces, but it was not addressed in this study.

Finally, a longitudinal study considering the long-term treatment outcome is recommended to investigate the quality of treatment decision making. Nonetheless, the fact that several parameters can interact with treatment decisions is an important issue that must be considered.

## Conclusion

This study shows that the medical management pathway for demographic, anthropometric, and radiographically comparable patients is mainly related to the reduced functionality of subjects selected to undergo TKR. This result can easily be assessed in clinics, helping medical doctors standardize the decision-making process in different hospitals. However, results suggest that age, BMI, and sex are confounding factors in treatment decisions, preventing the description of a fixed threshold that allow to discriminate between the kind of therapy.

Mechanical factors are limited to joint moments and interact with age and BMI. Different moments might be related to the different positions of the joints, and therefore different contact points at the cartilage level. These data should be evaluated in future studies, together with the kinematics of the subjects, to verify the stiffness of these patients (Zeni and Higginson, 2009) and the relation to the perceived pain.

The effects on gait are complex, and comprehensive analysis must consider all the factors together; otherwise, a study might result in nonreproducible or not comparable results. For instance (Astephen et al., 2008) presented a list of significant parameters that were not significant in this study. They show how the variability of the gait profile decrease with the increasing severity of OA. This is something that in our study was related to age and BMI. Variation of the selected parameters along the gait cycle might be related to several factors that might or not be related to severity of OA. This makes the study of variability through tools like time series analysis of particular interest to understand the pathology.

In summary, the message of the study is that the relation among the studied factors is not linear. Therefore, to describe the final treatment decision, objective gait descriptors should be combined with the aforementioned factors, because the clinical situation of the subject might require different evaluation threshold.

## Data Availability

The original contributions presented in the study are included in the article/Supplementary Material, further inquiries can be directed to the corresponding author.

## Abbreviations

BMI, Body mass Index; KL, Kellgren–Lawrence; KOA, Knee osteoarthritis; MANOVA, Multivariate Analysis of Variance; OA, Osteoarthritis; SYSADOAs, Symptomatic slow action drugs for osteoarthritis; TKR, Total knee replacement.
