# Supplementary material for: Relationship Between the Choice of Clinical Treatment, Gait Functionality and Kinetics in Patients With Comparable Knee Osteoarthritis
Source: Front Bioeng Biotechnol. 2022 Mar 11;10:820186. doi: 10.3389/fbioe.2022.820186 (PMC8962661; doi:10.3389/fbioe.2022.820186)
Supplement: Supplementary file 3 [file DataSheet3.docx]

# Background information

Contents

[Background information 1](#_Toc85012436)

[Generalization to other populations and clinics. 2](#_Toc85012437)

[Helen Hayes marker protocol and inverse dynamics 2](#_Toc85012438)

[Visual Consistency evaluation 3](#_Toc85012439)

Generalization to other populations and clinics.

*It does not exist at the moment any generalized guideline that can help clinicians to perform uniform treatments in different clinics, even within the same country. Different rates of TKR surgery have been described in different areas and countries. In Spain, the population-adjusted rate ranges from 26 to 702 per 100.000 population over the different regions*^1^*. Data analysis from 27 countries belonging to the Organization for Economic Co-operation and Development (OECD) showed also elevated differences in the age-standardized incidence rates that vary from 48.5 (Poland) to 292.1 (USA)*^2^*. These data highlight the importance to describe homogeneous indication criteria for TKR. The results of our study show an association between specific gait patterns and the decision to treat an OA patient through TKR. Therefore, we propose to consider the corresponding gait factors and their interactions with others to uniformize the treatment decision.*

*Arguably, our conclusions based on the statistical analysis of our data are influenced by the particular clinical decision tree used in our hospital. At the Hospital of reference, core treatment is initially based on non-pharmacological measures such as access to adapted high-quality information, advice for weight loss, exercise and the use of appropriate footwear. When this is not enough, depending on the patient's phenotype, the possibility to treat with oral or topical NSAIDs or/and intra-articular treatment is offered. In selected cases, opioids, duloxetine, and SYSADOAs are used. Patients are given a high-quality information which empowers her/him to share the decision. They are placed on the waiting list for TKR when they present an advanced radiological grade and when function and pain are considered to not being improving enough through the aforementioned nonsurgical management.*

## Helen Hayes marker protocol and inverse dynamics

*The protocol Helen Hayes with medial markers used in this study allowed for the measurement of rigid body kinematics. In particular, foot progression and dorsi-plantar flection, knee flexion-extension, abduction-adduction and internal external rotation and hip flexion-extension, abduction-adduction and internal external rotation.*

Table 1From Left to right: angle of rotation for Ankle, Knee and Hip. Flexo-extension in red, Internal-External rotation in green and abduction adduction in blue.

| 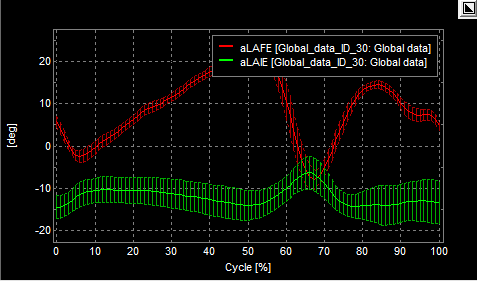 | 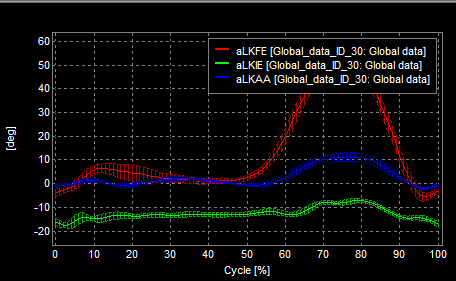 | 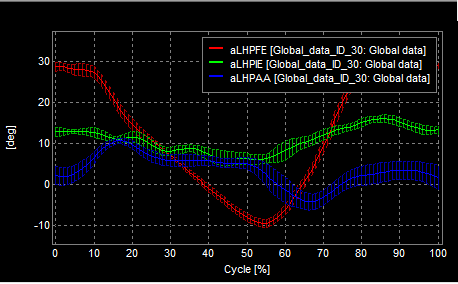 |
| --- | --- | --- |

*The relative position of the rigid body segments, together with the ground reaction forces and moment, are applied to an inverse dynamic process implemented in the software smart Analyzer, allowing the calculation of contact forces and internal rotation moments, in particular compression, medio-lateral and anterior–posterior shear forces, and flexion–extension, abduction–adduction, and internal-external rotation moments. Ankle dorsi–plantar flexion moments and medio-lateral forces were also computed.*

Table 2 From left to right, first row: contact forces for Ankle, Knee and Hip. Medio-lateral in red, Anterior-posterior in green and compression force in blue. From left to right, second row: rotation moments for Ankle, Knee and Hip. Flexo-extension in red, Abduction-adduction in greem and internal-external rotation in blue.

| 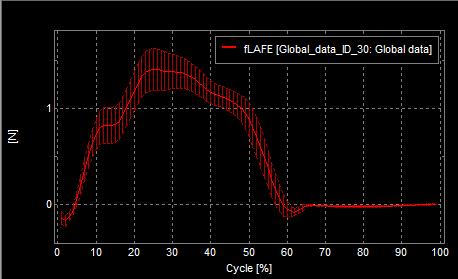 | 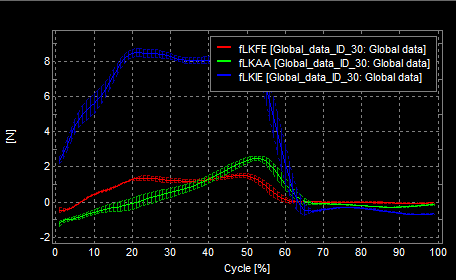 | 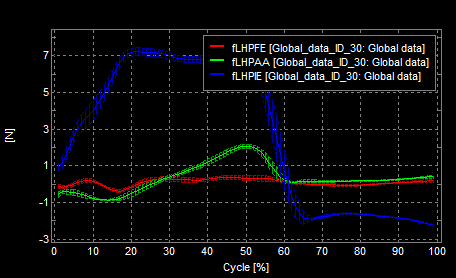 |
| --- | --- | --- |
| 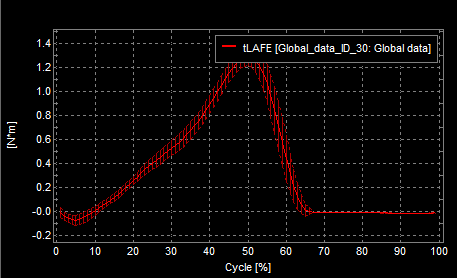 | 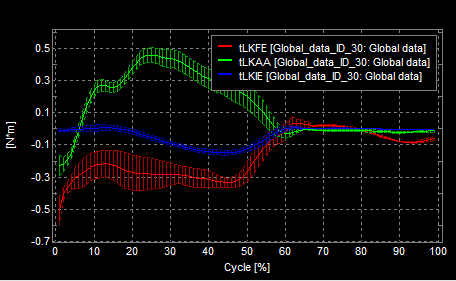 | 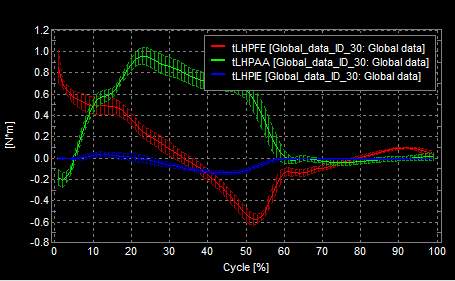 |

## Visual Consistency evaluation

*Human gait has intrinsic variability and under certain circumstances this variability can be higher in healthy subjects than in pathological ones. For this reason, it is important to look at the general behavior of the subject to identify possible outliers. The visual inspection has the aim to identify if specific trials are falling outside the variability of the analyzed subject. In the following example the ground reaction forces of two subject are reported. In the second one an example of removed discrepancy is reported.*

Table 3 The three ground reaction forces of two subjects are reported. In the first one all the trials are comparable while in the second one an outlier is reported.

| 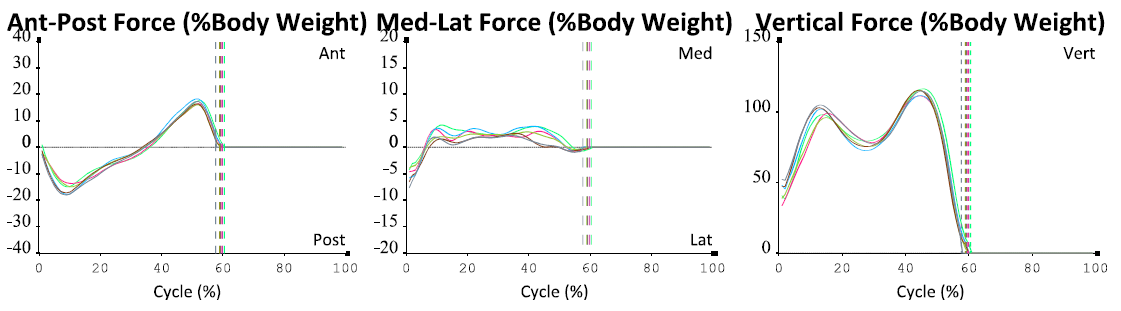 |
| --- |
| 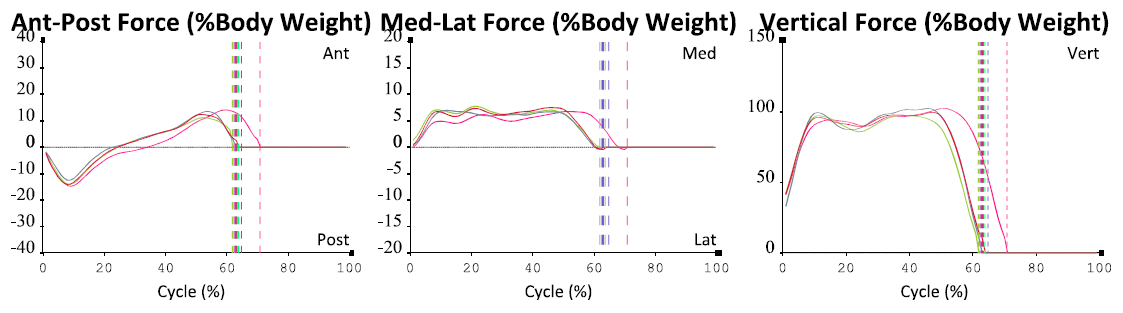 |
