# Supplementary material for: Relationship Between the Choice of Clinical Treatment, Gait Functionality and Kinetics in Patients With Comparable Knee Osteoarthritis
Source: Front Bioeng Biotechnol. 2022 Mar 11;10:820186. doi: 10.3389/fbioe.2022.820186 (PMC8962661; doi:10.3389/fbioe.2022.820186)
Supplement: Supplementary file 5 [file DataSheet2.docx]

# Power Analysis

*The reviewer is pointing out an important issue in the analysis of clinical data, underlining two different aspects: power analysis and dimension of the groups that the authors further comment below.*

*Power analysis: a priory test was performed. The study is based over the evaluation of three independent MANOVA analysis. To keep the global error withing 5% each MANOVA was performed using alpha value 0.018. Assuming an average effect size of 0.15 (Cohen’s 1977,1988) the power analysis would require a sample size of 96 volunteers to obtain an actual power of 0.84. With the actual number volunteers (87), a post hoc analysis of power shows a power of 0.78 for each of the performed MANOVA. This data refer to the time and spatial analysis, which result to be the most critical since it present only 3 response variable. In the case of Moments and Forces, only 64 volunteers would be required to obtain a power of 0.90 and 0.95 respectively. Analysis reported in the figure below.*

| 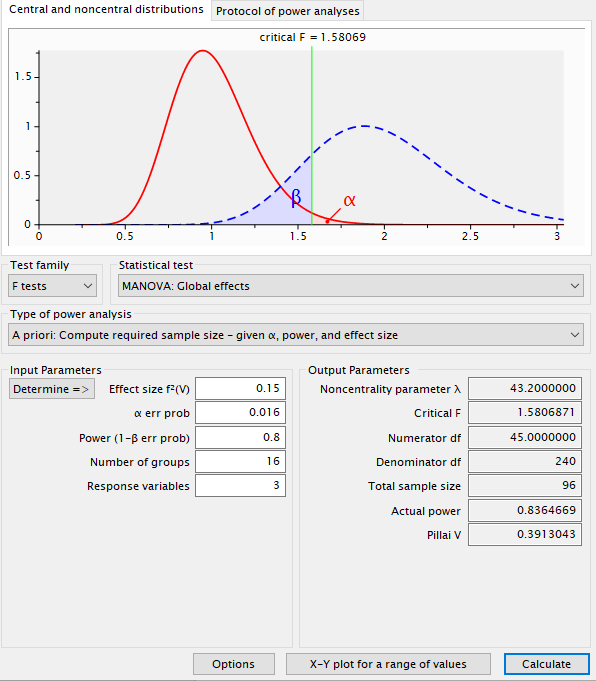 | 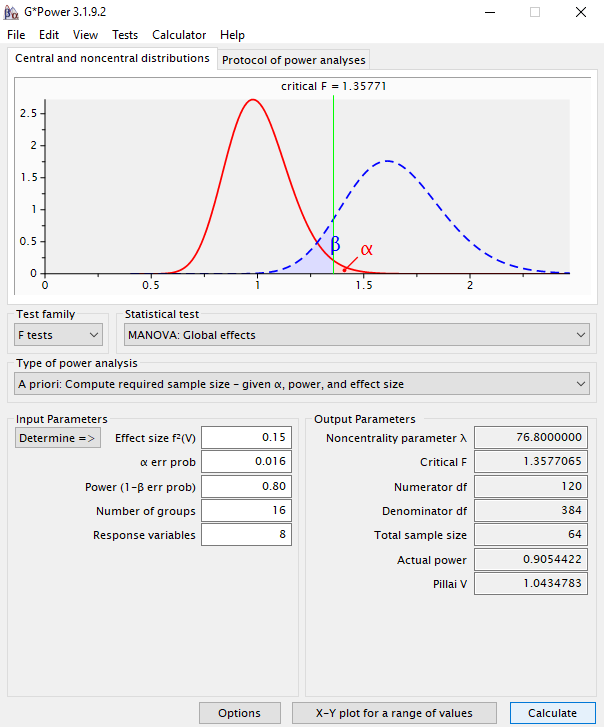 | 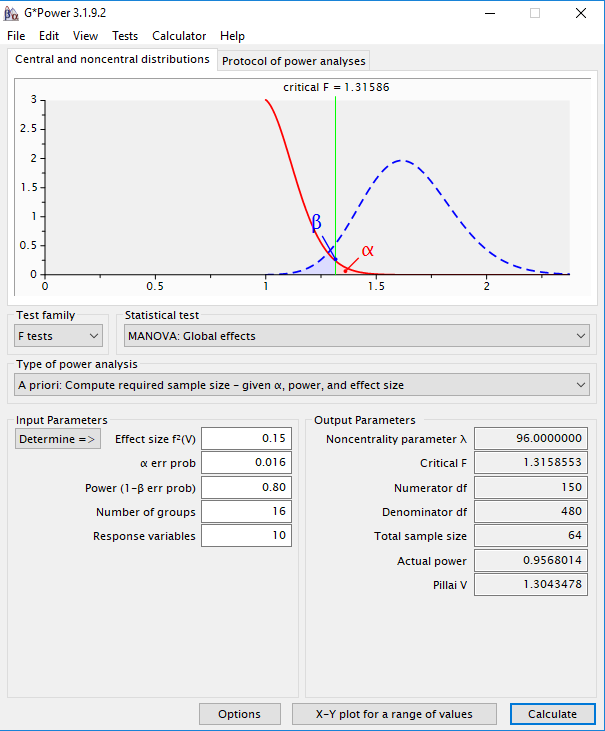 |
| --- | --- | --- |
| *Time and Space* | *Moments* | *Forces* |
