# Supplementary material for: Relationship Between the Choice of Clinical Treatment, Gait Functionality and Kinetics in Patients With Comparable Knee Osteoarthritis
Source: Front Bioeng Biotechnol. 2022 Mar 11;10:820186. doi: 10.3389/fbioe.2022.820186 (PMC8962661; doi:10.3389/fbioe.2022.820186)
Supplement: Supplementary file 6 [file DataSheet1.docx]

# Description of Supplementary Materials

The here presented document describes the supplementary materials related to the main work.

Three excel files are added, one for each of the category of variables analysed: 01) functionality analysis, 02) Forces analysis 03) Torques analysis.

The structure of each file is similar. The first Tab, named “raw data”, presents the original data collection of each anonymized subject. The multivariate tab reports the complete results of the multivariate ANOVA while the Univariate tab reports the single analysis for each of the variables analysed in the Multivariate ANOVA.

In Forces and Torques Univariates results are divided in “between” (analysis of the 4 main factors) and “within” (analysis of the repeated measures in the three time points).

Two supplementary text files were added as supplementary material. One related to the power analysis of the study and one for the relative background information.
